# Comparison of an unsupervised machine learning algorithm and surgeon diagnosis in the clinical differentiation of metopic craniosynostosis and benign metopic ridge

**DOI:** 10.1038/s41598-018-24756-7

**Published:** 2018-04-20

**Authors:** Min-Jeong Cho, Rami R. Hallac, Maleeh Effendi, James R. Seaward, Alex A. Kane

**Affiliations:** 1 Department of Plastic Surgery, UT Southwestern School of Medicine, Dallas, TX United States; 2 Analytical Imaging and Modeling Center, Children’s Medical Center, Dallas, Texas United States; 3 Texas Tech University Health Science Center School of Medicine, Lubbock, TX USA

## Abstract

Metopic suture closure can manifest as a benign metopic ridge (BMR), a variant of normal, to “true” metopic craniosynostosis (MCS), which is associated with severe trigonocephaly. Currently, there is no gold standard for how much associated orbitofrontal dysmorphology should trigger surgical intervention. In our study, we used three-dimensional (3D) curvature analysis to separate the phenotypes along the spectrum, and to compare surgeons’ thresholds for operation. Three-dimensional curvature analyses on 43 subject patients revealed that the mean curvature of mid-forehead vertical ridge was higher for patients who underwent operation than those who did not undergo operation by 1.3 m^−1^ (p < 0.0001). In addition, these patients had more retruded supraorbital areas by −16.1 m^−1^ (p < 0.0001). K-means clustering classified patients into two different severity groups, and with the exception of 2 patients, the algorithm’s classification of deformity completely agreed with the surgeons’ decisions to offer either conservative or operative therapy (i.e. 96% agreement). The described methods are effective in classifying severity of deformity and in our experience closely approximate surgeon therapeutic decision making. These methods offer the possibility to consistently determine when surgical intervention may be beneficial and to avoid unnecessary surgeries on children with benign metopic ridge and associated minimal orbitofrontal deformity.

## Introduction

Metopic craniosynostosis (MCS) is a challenging condition to diagnose, manage, and treat due to the wide spectrum of presenting deformity. In large historical clinical series, metopic craniosynostosis accounts for 3–4% percent of all single suture craniosynostosis, but recently there has been a marked epidemological increase in its prevalance to as high as 28%^1–3^ of all surgically treated synostosis cases. The etiopathogenesis of this shift is unknown but it has been speculated that the reasons are multifactorial, including an increasing proportion of syndromic patients, and advanced paternal age^4^. However, some practitioners are concerned that this phenomenon may be secondary to over-diagnosis and concomitant decision to offer operation on benign metopic ridge (BMR), which is present in 10–25% of infants as a variant of normal^5^.

Currently, there are no level I or II evidence-based treatment algorithms for MCS which incorporate classification of severity into decisions regarding indications for surgery versus conservative treatment^6^. Surgeons have used different diagnostic and treatment criteria for patients with MCS^7,8^. It is not surprising then, that surgeons have different thresholds for operative intervention, and this has caused the management of milder forms of metopic craniosynostosis to become a controversial topic.

There has not been a study to evaluate and quantify surgeons’ thresholds for operative intervention in metopic craniosynostosis. In our study, we have used 3D curvature analysis to classify different phenotypes along the spectrum of metopic craniosynostosis, and to correlate surgeons’ comparative thresholds for operation at one tertiary care craniofacial center.

## Results

The study population consisted of 43 patients who were evaluated and treated by 5 surgeons from 2010–2015. Of the 43, 16 patients underwent surgical treatment while the other patients were managed conservatively after an initial evaluation.

### Non-surgically Treated Group

The mean age of patients was 8.3 months at the time of initial evaluation, and 37% of this group was male. The average gestational age was 37.9 weeks. 30% of this group were born via cesarean, 4% via non-spontaneous vaginal delivery, and 30% via spontaneous vaginal delivery.

### Surgically Treated Group

The mean age of patients was 5.9 months at the time of initial evaluation and 9.1 months at the time of operation. 75% of this group was male with average gestational age of 38.1 weeks. 50% patients were born via cesarean section, 12% via non-spontaneous vaginal delivery, and 31% via spontaneous vaginal delivery. Of the 16 patients, 31% were treated with metopic strip craniotomy while the other 69% were treated with bifrontal craniotomy and fronto-orbital reconstruction.

In comparison to the non-surgically treated group, the percentage of males in the surgically treated group was higher. Although it’s an interesting finding, it is difficult to determine the reason for the difference given that this is a retrospective study.

### Regions of interest

Table 1 shows the average mean curvature for the mid-forehead vertical strip and right/left supraorbital regions. The mean curvature of the mid-forehead vertical strip for the surgically treated group was 39.0 ± 6.9 m^−1^, and 27.7 ± 4.6 m^−1^ for the non-surgically treated group with a difference of 11.3 m^−1^ (the number following the symbol “±” indicates standard deviation). There was a significant difference between these two groups (p < 0.0001). The mean curvature of mid-forehead vertical strip for the severe abnormality cluster was 38.6 ± 7.1 m^−1^, and 28.0 ± 4.9 m^−1^ for the mild abnormality cluster, which was also significant (Table 1).

**Table 1:** Average mean curvature for the two regions (mid-forehead strip and right/left lateral orbital areas).

Average mean curvatures		
	Mid-forehead strip (m^−1^)	Right/left lateral orbital areas (m^−1^)
Surgically treated group	39.0 ± 6.9	−5.9 ± 5.7
Non-surgically treated group	27.7 ± 4.6	10.2 ± 6.3
Severe abnormality cluster	38.6 ± 7.1	−6.7 ± 4.7
Mild abnormality cluster	28.0 ± 4.9	10.6 ± 5.5

The mean curvature of right/left supraorbital regions for the surgically treated group (−5.9 ± 5.7 m^−1^) and non-surgically treated group (10.2 ± 6.3 m^−1^) were statistically different (p < 0.0001). The mean curvature of orbital areas for the severe cluster was −6.7 ± 4.7 m^−1^, and 10.6 ± 5.5 m^−1^ for the mild cluster, which was also statistically significant (Table 1).

### K-means cluster analysis

**Figure 1:**
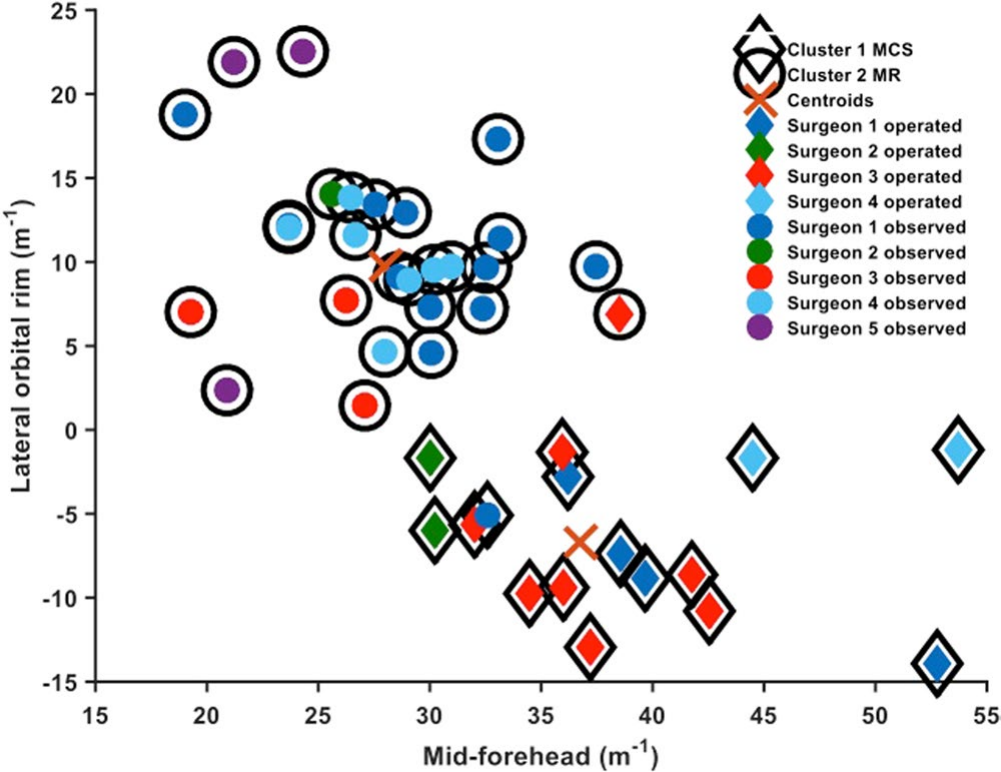
K-means cluster analysis of mean curvature for all patients. The two clustered groups represent algorithmically determined BMR patients (cluster 1; with surrounding black circle), and algorithmically determined MCS patients (cluster 2; with black diamond surrounding). Colored interior circles represent individual patients that underwent conservative management, with colors representing different surgeons. Colored interior diamonds represents operatively treated patients, with colors representing different surgeons.

**Figure 2:**
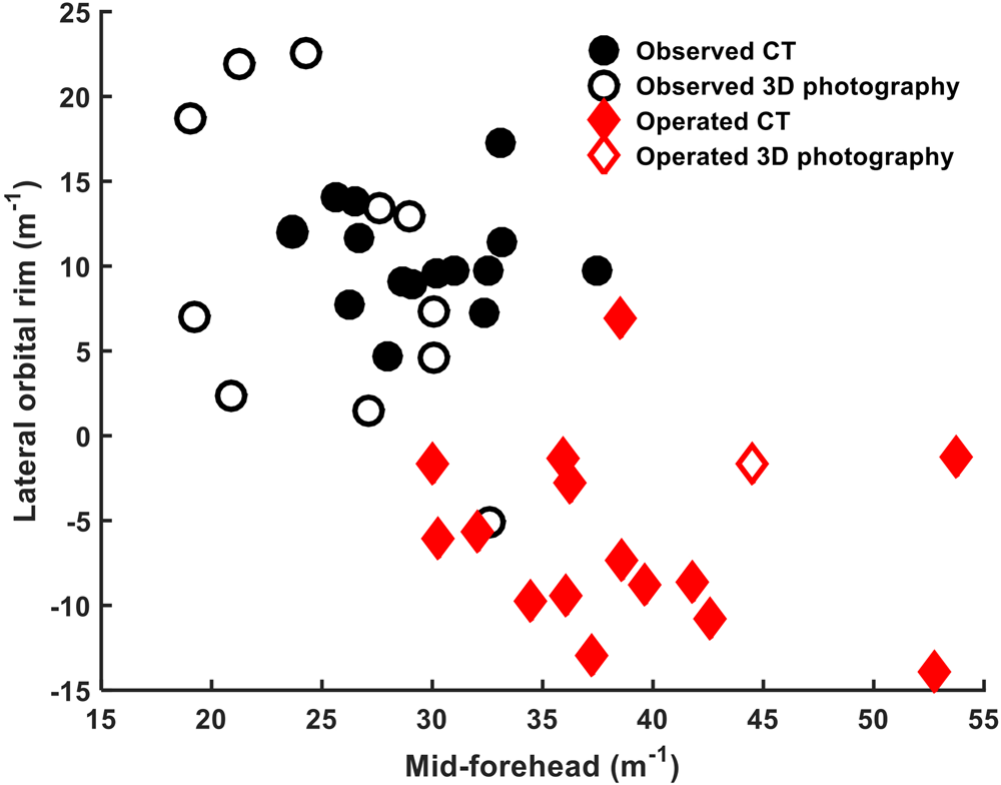
Demonstration of algorithmic outcome by imaging type (CT versus Sterephotogrammetric image).

The cluster analysis classified the 43 patients into two phenotypes: 27 corresponding with BMR and 16 corresponding with MCS based on the mean curvatures of the segmented mid-forehead and left/right supraorbital regions (Figs 1 and 2). There was 96% agreement between the automated algorithmic clustering and surgeons’ decision to operate or conservatively manage the children. Of the 43 patients, 2 patients had disagreement between the algorithm and clinical management. One patient who was classified as BMR by the algorithm underwent surgical intervention, and one patient who was classified as MCS by the algorithm was conservatively managed (Figs 1 and 2).

## Discussion

Determining the degree of orbitofrontal dysmorphology required to trigger surgical intervention in children with metopic deformities has been a controversial topic for craniofacial surgeons. The metopic suture is unique in that it is the only suture whose fate is to close, and it does so in the first year of life^9^. As the suture closes, many children have a metopic ridge and some degree of associated orbitofrontal deformity. Unlike other single-suture synostoses, differentiating benign metopic ridge, which is accompanied by a lesser degree of orbitofrontal dysmorphology, from full-blown metopic craniosynostosis can be difficult due to lack of gold-standard diagnostic and treatment guidelines. Therefore, the management of patients who fall in the middle of the spectrum has caused heated debate in the craniofacial community. In one study, Yee *et al*. demonstrated an almost 50:50 divide between operative (53.1%) and observational (46.9%)^10^ preferences in their survey. This survey clearly demonstrated how practitioners may disagree significantly in severity classification.

No level I or II evidence-based guidelines exist to classify severity of deformity and to recommend management of these deformities^6^. The literature provides several methods to quantify the severity of metopic craniosynostosis using CT scan measurements of the intercoronal distance, lateral orbital wall distance, medial wall protrusion^11^, the endocranial bifrontal angle^12^, temporal deformity^13^, and trigonocephaly severity indices^14^.

In this study, we used a novel curvature analysis to automatically classify deformity as being either consistent with BMR or MCS. In the 43 patients evaluated by 5 surgeons over a 5 year period, the mean curvatures of the segmented mid-forehead vertical strip and lateral supraorbital areas was significantly higher in the surgically treated group, which strongly suggests that these areas are key to treatment decision making. These findings are in agreement with the classical teaching of offering operation to patients with more significant supraorbital flattening, and other quantitative studies that have demonstrated greater orbitofrontal narrowing and retrusion of the lateral orbital rims^11,12,14^.

These methods compactly quantitate the severity of the spectrum of patients treated at our clinic based upon the mean curvatures of two easily specified orbitofrontal surface features. Furthermore, they provide a convenient means to correlate severity to treatment thresholds/preferences of the surgeon. When considered as a group, the 5 surgeons seeing these patients had relatively similar thresholds for operation. In patients with similar mid-forehead ridging, surgeons operated on patients who had more retrusion of lateral orbital rims. These surgeons in this study are all board-certified plastic surgeons and focus their practice on craniofacial surgery, with the range of post-fellowship experience being from 4 to 26 years, including three junior surgeons (<10 years of practice) and two senior surgeons (>10 years of practice).

However, the 2 instances where the algorithm and the treatment were discordant offer an interesting window into practice. In order to better understand why the 2 patients had a disagreement between the algorithm and surgeons’ decision to operate or conservatively manage the patient, we asked the 4 surgeons at our craniofacial center to review 7 photographic views of the 2 patients’ clinical photographs (frontal, lateral, basal, top, and posterior views). For the patient who was classified as BMR by the algorithm that underwent surgical intervention, all 4 surgeons who saw the photos determined that this patient had BMR. The question then arises as to why the child underwent surgical intervention. Possibilities include misdiagnosis, or possibly that the photographs do not offer the same information as a clinical examination does. Upon chart review, this patient did have developmental delay at the time of evaluation, which may have influenced the decision to offer surgery. In addition, parental concern could have impacted the decision to operate as it is ultimately a matter of communication between parent and surgeon as to whether the child should receive an operation. For the patient that the algorithm classified as MCS but was conservatively managed, 2 surgeons who viewed the photos believed that the patient had MCS while the other 2 surgeons believed that the diagnosis was BMR. This phenomenon mirrors the finding by Yee *et al*., where providers were split 50:50. In addition, it demonstrates the difficulty of differentiating phenotypes of metopic suture closure, and the benefit of using quantitative assessment in diagnosis. It should be again stressed, however, that there were only 2 disagreements between algorithmic classification and treatment offered at our institution.

Recently, there has been a reported three-fold increase in the prevalence of metopic craniosynostosis from one in 15,000 births to one in 5,000 births. While much is not known about the etiopathologic basis of the observed increase, it is prudent to keep in mind that there is historical precedent for large surges in the diagnosis of craniosynostosis. There was a period in the relatively recent past when many hundreds of patients received unnecessary craniotomies for wrongly diagnosed lambdoid craniosynostosis, when the true diagnosis was deformational plagiocephaly with “sticky” lambdoid suture. This phenomenon led to CDC (Centers for Disease Control and Prevention) investigation^15,16^. It is certainly possible (although not provable in this study) that the epidemiological increase in metopic craniosynostosis is related to operating on patients with benign metopic ridge.

Given the radiation associated with CT and increased the increased availability of stereophotogrammetric images^17–20^, we believe that curvature analysis can be used as means to communicate among craniofacial surgeons, compare patient’s severity and surgical thresholds across institutions. Moreover, a database of patients from different institutes could be created, and provide a severity spectrum of metopic craniosynostosis between many institutions. This technology could be impactful in forging consensus with respect to indications for operation and thereby could confer protective benefit in preventing unnecessary craniotomies and assuring uniformity in treatment.

In conclusion, we demonstrate that curvature analysis is a useful tool for automatically classifying deformity with potentially very meaningful diagnostic impact. The surgeons in our center have similar thresholds for managing patients conservatively or surgically, regardless of duration of experience and differing clinical training. The retrusion of the lateral supraorbital areas are key factors in separating benign metopic ridge and metopic craniosynostosis, and 3D curvature analysis is equally applicable to CT and stereophotogrammetric images. These methods offer the potential for objective diagnosis and treatment guidance, which could reduce unnecessary surgical interventions.

## Methods

This study was approved by the Institutional Review Board (IRB), and it was carried out in accordance with IRB guidelines and regulations. The IRB approved a waiver of informed consent given that our study is a retrospective review. After obtaining Institutional Review Board (IRB) approval, the records of patients presenting with concerns related to metopic deformity to our tertiary craniofacial center between the years of 1995 and 2015 were reviewed.

**Figure 3:**
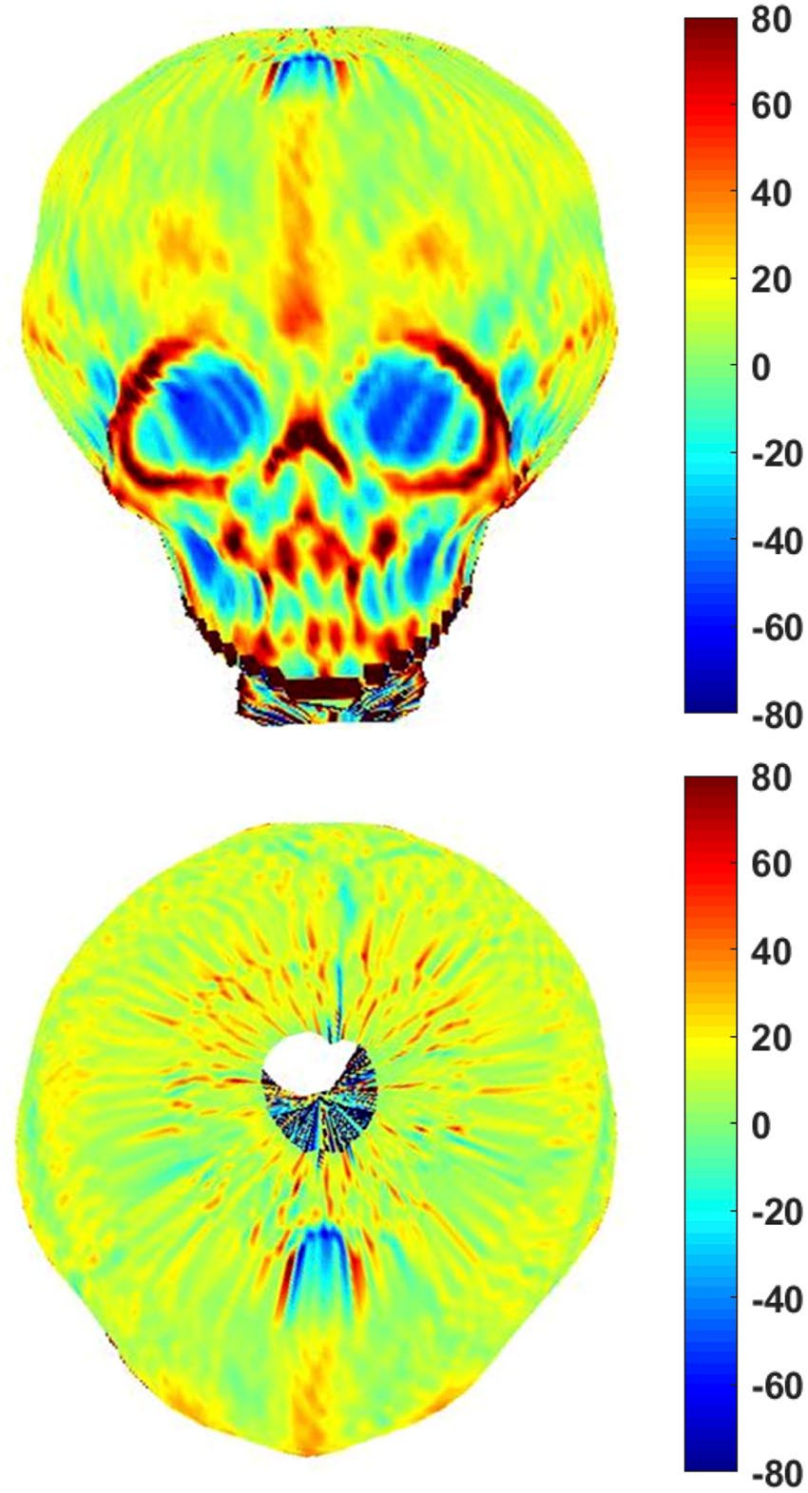
3D curvature analysis of patient with BMR managed conservatively.

**Figure 4:**
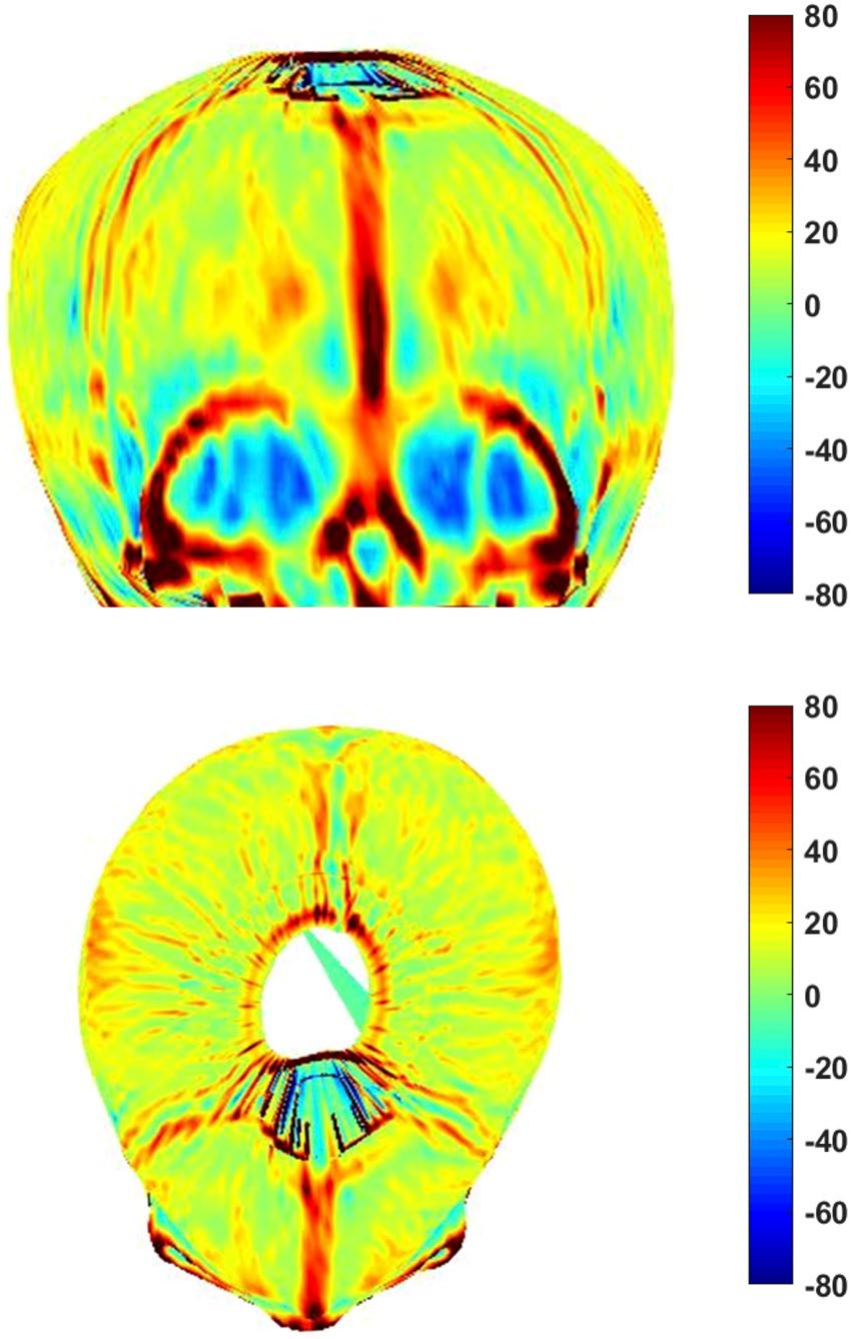
A 3D curvature analysis of patient with MCS managed operatively.

All patients who underwent whole head computational tomography (CT) or stereophotogrammetry imaging (3dMD face system, Atlanta, GA) as a part of their diagnostic workup were included. CTs were obtained in cases whether the diagnosis is questionable based solely on physical examination. A total of 43 patients were identified for the study: 27 patients who were managed conservatively (Fig. 3) and 16 patients who underwent operation (Fig. 4).

CT images of patients were converted into 3D images using the methodology described in our pilot study^21^, which tested the validity of 3D curvature analysis in differentiating and classifying different phenotypes of metopic suture closure along the spectrum. Using this methodology, images were downloaded from hospital PACS (Picture Archiving and Communication System) in DICOM (Digital Imaging and Communications in Medicine) format. Mimics 10.01 software (Materialise, Leuven, Belgium) was used to reconstruct three-dimensional (3D) surface images by removing soft tissues from the bony structures using thresholding techniques with digital closure of any surface bone gaps. Images were then converted to stereolithography (STL) file format. Curvature analysis was performed using custom algorithms written in MATLAB (MathWorks, Massachusetts, USA). 3dMD stereophotogrammetric images were converted to STL format and curvature analysis was performed.

**Figure 5:**
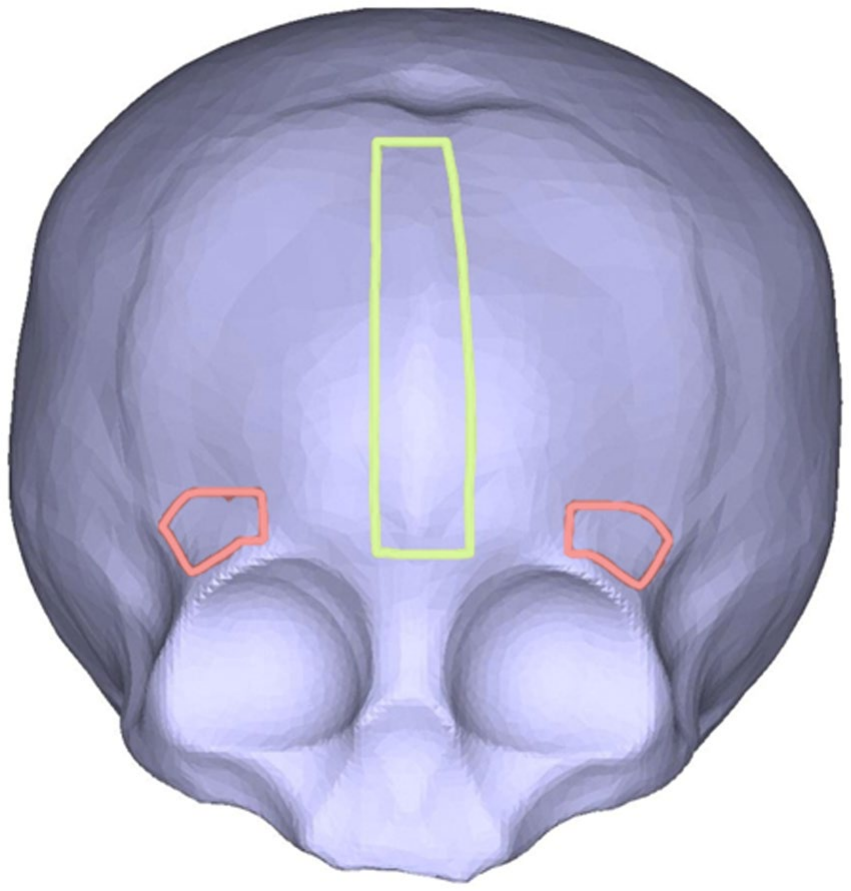
Three segmented regions of interest: mid-forehead strip (yellow), right/left lateral supraorbital areas (pink).

Three regions of interest were interactively segmented from the prepared STL files by a single individual (M.J.C). These 3 regions were selected to differentiate between phenotypes of metopic craniosynostosis based upon orbitofrontal dysmorphology. These regions included a single vertical mid-forehead strip overlying the metopic ridge, and paired right/left horizontally oriented supraorbital strips. The mid-forehead metopic strip was defined as a rectangular region over the metopic suture, measuring 10 mm width and extending from the subject’s glabella to anterior fontanelle (Fig. 5). Right/left supraorbital areas were defined above the superior orbital rim on each side, measuring 10 mm width and extending the length of subject’s lateral to mid superior orbital rim (Fig. 5).

K-means cluster analysis was used to automatically classify subjects into groups of similar characteristics based on the curvatures of the three segmented regions. The analysis automatically generated two groups of subjects based on the anatomical characteristics of these regions: one corresponding with BMR, and another corresponding with MCS. The outcome of this automated clustering was then compared with the actual clinical treatment received (i.e. conservative vs. operative).
